# The effect of infantile colic training given to parents on the neonatal infantile colic level and crying duration

**DOI:** 10.1038/s41598-025-34344-1

**Published:** 2026-01-28

**Authors:** Serap Özdemir, Gizem Yilmaz, Nisa Nur Çelik, Fatmagül Bayram

**Affiliations:** 1 Department of Pediatric Nursing, Faculty of Health Sciences, Gaziantep University, Gaziantep, Turkey; 2 Department of Nursing, Yusuf Serefoglu Faculty of Health Sciences, Kilis 7 Aralik University, Kilis, Turkey

**Keywords:** Infantile colic, Training, Crying, Newborn, Diseases, Health care, Medical research

## Abstract

Infantile colic is an important problem encountered during the first months of life. This study investigated the effect of infantile colic training provided to parents on the severity of neonatal infantile colic and the duration of crying. The research used a one-group pretest–posttest design. The study was conducted among the mothers and fathers of newborns who attended a Family Health Center between May and October 2024 and were diagnosed with infantile colic. A power analysis was conducted, and the sample consisted of 60 parents. Parents received infantile colic training in a single session, and its effectiveness was evaluated at two different times. Data were obtained via a questionnaire that included a sociodemographic form and the Infantile Colic Scale. The crying times of the newborns were recorded based on the information provided by the parents. Statistically significant differences in the Infantile Colic Scale scores were not found in mothers after the training; however, significant differences were observed in the first post-education assessment of fathers and between the parents’ first and second assessments. The mean duration of infant crying after the training was found to have a high effect size. After the training, it was found that the severity of newborn infantile colic and the duration of crying decreased. This training is an effective intervention for neonatal infantile colic.

**Supplementary Information:**

The online version contains supplementary material available at 10.1038/s41598-025-34344-1.

## Introduction

Infantile colic (IC) can have serious consequences for both the infant and the family. This condition not only disrupts the infant’s development but also poses a critical threat to the family’s lifestyle. IC is a common and distressing condition in infancy, of unknown etiology, characterized by paroxysmal attacks. These attacks involve excessive crying that is very difficult to stop, often accompanied by signs such as clenched fists, abdominal rigidity, flatulence, and pulling of the legs toward the abdomen^1–3^. According to Wessel et al.^4^, IC is defined as unexplained and uncontrollable crying spells with onset in the first seven days of life, lasting more than three hours daily and three days per week, typically occurring in the afternoon and/or evening. Additionally, infantile colic is evaluated according to the ROME IV diagnostic criteria. These are defined as recurrent and prolonged periods of distress/crying/irritability in infants from birth to 5 months of age, which cannot be prevented or resolved by caregivers, from the onset to the end of symptoms^5^. Depending on the diagnostic criteria used, it is seen in 5–73% of the neonatal period, especially in the first three months of life. It usually appears in the second week of life and can persist for up to 4–6 months^2,6,7^. Linking IC to developing functional disability bowel syndrome later in life has been reported. This makes IC a problem that needs to be addressed in the newborn period^8^. Its onset in the neonatal period may cause different problems for the baby and the parents^9,10^. Frequent and prolonged crying episodes can cause significant behavioral problems with infant sleep, rest, feeding, and mother-infant bonding^1–3^. Moreover, caring for an infant with colic can be challenging enough to disrupt the quality of life of the whole family. Mothers in particular may experience fatigue, helplessness, inadequacy, poor concentration^9^, insomnia, loss of confidence^3^, exhaustion, anxiety, and breastfeeding problems^2^. Indrio et al.^8^ reported that behavioral problems were linked to higher parenting stress scores in children with a history of IC. The exact cause of IC is not known, and some neurodevelopmental, gastrointestinal, and psychosocial factors are thought to play a role^1,7^. Because the exact cause is unknown, there is no definitive treatment. In recent years, several symptomatic treatments have been proposed. These symptomatic treatments include medication, diet, and behavioral approaches^2,11,12^. Drug treatments are not recommended because they are expensive and have side effects. Therefore, behavioral approaches come to the forefront of the IC method^6,13–16^.

Parents often use behavioral approaches to reduce IC symptoms and comfort the baby, such as paying attention to the mother’s diet^9^, patting the baby’s back, massage/exercise^17^, rocking the baby, positioning the baby^18^, swaddling the baby, taking the baby for a ride^19^, abdominal massage and hot pet application, giving herbal teas^12^, singing songs/lullabies, and running a blow dryer/vacuum cleaner^2,20^, acupuncture^21^, reflexology^19,22^, karyosacral therapy^23^, pacifier use^20^, kangaroo care, white noise^24^. In these behavioral responses; babies cry less, their tension decreases, and they sleep more with the decrease in colic severity, and these situations positively affect the comfort of babies and their families^18,21,23^.

IC management should include new assessments such as infant and parent quality of life, sleep duration, and impact of illness on parents^8^. The educational and counseling duty of pediatric nurses is important in teaching the onset of IC, its physiology, its impact on the baby, and the methods to be applied to relieve the baby and parents^21,25,26^. In a study examining the effect of education provided to mothers on the IC method, it was reported that the rate of neonatal IC cases in the sample was 9.8%^27^. No other scientific study was found on family education in managing IC starting from the neonatal period. With this study, our goal was the creation of literature on this topic. It was thought that early training of nurses and observation of their effects would contribute to health services, especially for families. Therefore, this study aimed to evaluate the impact of training newborns to avoid colic in parents on the level of neonatal infantile colic and crying duration.

*Hypotheses*

### H0

*Infantile colic training given to parents does not affect the level of neonatal infantile colic.*

### H1

*Infantile colic training provided to parents reduces the level of neonatal infantile colic.*

### H2

*Infantile colic training given to parents reduces the duration of crying caused by neonatal infantile colic.*

## Methods

### Data source & study design

The research has a one-group pretest–posttest design. The study was conducted among the parents of newborns who applied to the Family Health Center between May and October 2024 and were diagnosed with infantile colic. The population consisted of parents of newborns (newborns who completed the first 15 days of life) who came to the Family Health Center for immunization or neonatal follow-up and had a diagnosis of infantile colic from the specialist pediatrician. To determine sample size, power analysis was performed using G*Power (v3.1.9.7). In the power analysis, the sample size was calculated to be 34 with α = 0.05, effect size = 0.50 (medium effect size), and power 80%^28^. However, with the assumption that there might be missing data, the sample was increased by approximately 50% and completed with 60 parents (30 mothers, 30 fathers). Inclusion criteria were being the parents of a newborn diagnosed with infantile colic by a pediatrician on the 15th day after birth and agreeing to participate in the study; however, parents who withdrew from the study during the study period, newborns with a congenital disease or anomaly, a history of intensive care, or a disease related to the gastrointestinal system were excluded.

### Data collection tools

*Questionnaire form* Developed through a literature review by researchers^1,3,22^. It is a 21-question form that includes parental sociodemographic characteristics and newborn nutrition.

### Infant colic scale (ICS)

Ellet et al.^29^ developed for the diagnosis and assessment of colic. Çetinkaya and Başbakkal^30^ investigated the validity and reliability of the scale in Turkey. The scale is composed of 5 sub-scales and 19 questions. Scale items are rated on a Likert scale from 1 to 6. Negative statements in the scale (questions 3, 7, 8, 9, 13, 14, 15, 17, 19) are reverse-coded. According to the scale evaluation, a low mean score indicates that colic complaints are decreasing, while a high mean score indicates that they are increasing. In the Turkish adaptation, Cronbach’s alpha was 0.73. In this research was 0.76.

### Training content

#### What is IC?

It is defined as crying episodes that are unexplained and uncontrollable which is occur during bouts from the 15th day after birth, such as clenching of the baby’s fists, hard abdomen, flatulence, pulling the legs to the abdomen, and crying bouts that are very difficult to stop and excessive (more than 3 h per day and 3 days per week, and particularly occurring in the afternoon and/or evening). If you observe these behaviors in your newborn, you should consult a doctor. At the doctor’s visit, your baby should be diagnosed with IC^6,13^.

#### Who gets IC?

It is known that male babies are more likely to be diagnosed with IC than female babies. If your baby is a boy, the risk of the disease is higher^1,3^. The younger the baby’s age (in days and months) and the older the mother, the higher the baby’s risk for this condition. Your baby is at risk for this disease if they are bottle-fed mixed (breast milk and formula)^1^. Risk factors for this disease include lactose intolerance. For your baby diagnosed with cow’s milk use IC, if the mother and/or father or a regular household member has a smoking habit, they should quit^7,14^.

#### What home remedies are available for IC management?

If your baby is fed with formula, it is recommended to use formula with reduced lactose content. In this case, you should know that the crying time of babies is shortened^11^. Cow’s milk formulas are known to increase the frequency of colic. You should make sure that the formula you use does not contain cow’s milk. In this case, you should know that the baby’s crying time is shortened^2,15^. The fact that the bond between the mother and the baby has not been sufficiently established, mental and psychological disorders of the mother, the mother’s first baby, and not getting enough support can trigger this disease in the baby. The mother should receive the necessary support (spouse, relative, person experienced in baby care) regarding her health^9^. IC pain wakes your baby up suddenly with crying and disrupts sleep patterns. Nursing mothers should avoid allergenic foods such as eggs, cow’s milk, legumes, dairy products, citrus fruits, cola, chocolate, and nuts. This diet should be maintained for two weeks, and if it is observed to work, the mother’s diet should continue without these foods^17,26^. In the sources, it is recommended that the mother consume herbal teas because they are beneficial. These are: cumin, lemon balm, fennel, mint, yarrow, dill, chamomile, licorice, and ginger^12^. It is recommended to breastfeed babies at night (due to the effect of the hormone called melatonin). You can use probiotics for your baby under the supervision of a doctor^16^.

#### What other practices can be used?

Some practices have been reported to help babies with infantile colic and reduce the duration of crying. These include: patting the baby’s back, therapeutic touch, massage/exercise, exposing the baby to the sound of a hair dryer, positioning, application of warmth to the baby’s abdomen, rocking, swaddling, exposing the baby to white noise, driving the baby in a car, singing songs/nursery rhymes, etc.^20,23,31^ are reported to be good for infantile colic.

### Data collection

The researchers collected the data in three steps.

**First,** after meeting and obtaining consent from the parents of newborns and infants diagnosed with IC, a questionnaire form including socio-demographic characteristics, ICS, and the crying times of the newborn in the last week was applied (pre-test). Afterward, the parents received theoretical training prepared in consultation with 10 experts (five academics in the field of pediatric health and disease nursing and five nurses) for 30 min in a quiet empty room. The educator (responsible author) also provided opportunities for questioning, discussion, and explanation using teaching strategies such as question-and-answer throughout the training. The training was given in the form of a presentation about what IC is, who has it, the practices that need to be done, and the practices that can be done at home. In addition, mothers and fathers were asked to record the average number of hours per day that the newborn cries at home without any specific reason (hunger, soiling, pain, illness, etc.).

**Second,** one week after the training, parents were asked to come to the Family Health Center for a follow-up visit. At the first post-training assessment, parents were administered the ICS and asked to record the duration of the baby’s crying during the previous week (post-test 1).

**Finally,** two weeks after the training, parents were asked to return to the Family Health Center for a follow-up visit. Parents were administered the ICS at the first post-training assessment and were asked to record their baby’s crying times during the previous week (post-test 2).

**Fig. 1:**
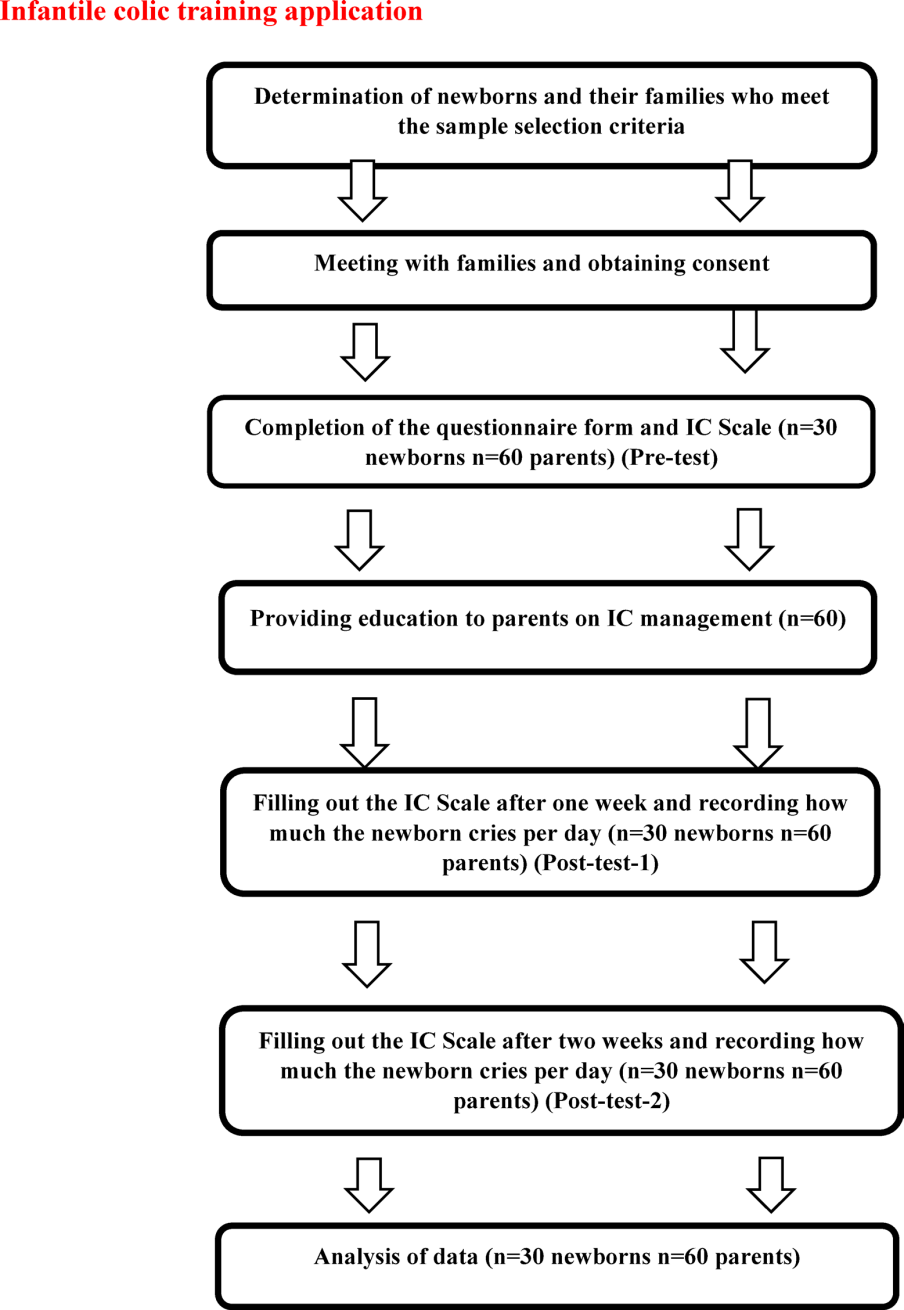
Research flow diagram.

Parents were interviewed a total of three times, including the training. This was because we wanted to evaluate the effectiveness of the training in the method of IC in the neonatal period. Infantile colic training application which is the research flow chart is shown in Fig. 1.

### Statistical analysis

Statistical Package for the Social Sciences (SPSS) v25.0 was used for data analysis. The Kolmogorov–Smirnov test was used to test for normality. Descriptive statistical analyses were conducted. Paired samples t-test was used to compare pre-test and post-test data. Student’s t-test was used to compare mean crying times, and Friedman’s test was used to compare three or more repeated measures (the first measurement, measured on days 7 and 15). Cronbach’s alpha coefficient was calculated for the reliability of the scale. Significance was accepted as *p* < 0.05 with a 95% confidence interval.

Group effect sizes were calculated using G*Power (3.1.9.7). The evaluation of effect size was performed according to Cohen. The effect size (Cohen’s f) was used to measure the magnitude of the experimental effect. It was suggested that f = 0.10 be considered “small”, 0.25 is “moderate”, and 0.40 is “large”. The larger the effect size, the more substantial the relationship between the four variables^32^.

### Ethical principles of research

Ethics committee approval (IRB: 06.02.2024-2024/01) from the university ethics committee. Permission was obtained from the institution where the study was conducted. The purpose and rationale of the study were explained to the parents. Verbal and written informed consent was obtained. Necessary permissions were obtained from those who conducted validity and reliability studies of the scales used. Throughout the research process, actions were taken in accordance with the principles outlined in the Declaration of Helsinki on Human Rights.

## Results

The characteristics of the newborns diagnosed with infantile colic and their parents are shown in Table 1.

**Table 1:** Socio-demographic characteristics of neonates and their parents (n = 30).

Characteristics	Mean ± SD	Min	Max
Age of mother (Years)	28.80 ± 6.55	19.00	42.0
Father’s age	33.80 ± 6.66	22.00	48.0
Newborn body weight (kg)	2887 ± 348	2100	3500
Newborn height (cm)	49.90 ± 1.1	48	52
Newborn head circumference (cm)	34.80 ± 0.65	34	36
Baby’s final body weight	3104 ± 359	2400	3700
Week of gestation	38.56 ± 0.64	38	40
Number of feedings per day	9.2 ± 2.0	2	13
Number of defecations per day	6.4 ± 1.1	3	8
Baby birth order	2.3 ± 1.3	1	6

Characteristics		n	%
Sex	Girl	18	60
	Boy	12	40
Mother’s Education Status	Literate	4	13.3
	Primary education	11	36.7
	High School	8	26.7
	University	7	23.3
Father’s Education Status	Literate	4	13.3
	Primary education	6	20
	High School	16	53.3
	University	4	13.3
Mother’s employment status	Yes	9	30
	No	21	70
Father’s employment status	Yes	29	96.7
	No	1	3.3
Family type	Nuclear family	29	96.7
	Extended family	1	3.3
The way the baby is born	Normal	17	56.7
	Cesarean	13	43.3
The baby’s diet	Breast milk	19	63.3
	Breast milk + water	3	10
	Breast milk + formula	2	6.7
	Formula	6	20
Bottle use status	Yes	10	33.3
	No	20	66.7
How to hold a bottle	Horizontal	8	80
	Vertical	2	20
Use of pacifier	Yes	16	53.3
	No	13	46.7
Application status for colic	Yes	24	40
	No	36	60
Applications for colic	Massage	15	25
	Gas drops	3	5
	Hot application	4	6.7
	Drinking water	2	3.3
Total		30	100

The mean maternal age was 28.80 ± 6.55 years, the mean paternal age was 33.80 ± 6.66 years, the mean newborn weight was 2887 ± 348, the mean height was 49.90 ± 1.1, the mean head circumference was 34.80 ± 0.65, the mean final weight was 3104 ± 359, the mean gestational week was 38.56 ± 0.64, and the newborns were the second child (2.3 ± 1.3). Newborns were breastfed on average 9.2 ± 2.0 times per day, and the mean number of defecations was 6.4 ± 1.1 times. In addition, 18 (60%) of the newborns in the study group were girl, 17 (56.7%) were born by cesarean section, 11 (36.7%) of the mothers had primary education, 16 (53.3%) of the fathers had high school education, 21 (70%) of the mothers were not working, 29 (96.7%) of the fathers were working, and 29 (96.7%) had a nuclear family structure. It was found that 19 (63.3%) of the newborns were exclusively breastfed, 20 (66.7%) did not use a bottle, 8 (80%) of the bottle users held the bottle horizontally, 16 (53.3%) used a pacifier, 36 (60%) of the parents did not use any colic remedy, and 15 (25%) of the remedy users used massage.

The compared of parents’ mean scores before and after IC training is shown in Table 2.

**Table 2:** Comparison of mean IC scores of parents before and after the training (n = 30).

ICS (total)		Mean ± SD (min–max)	Test^a^	*p*
Mothers	Pre-test (first day)	71.80 ± 10.80 (51–95)	− 2.019	0.530
	Post-test 1 (day 7)	71.76 ± 10.79 (52–95)		
	Post-test 2 (day 15)	70.86 ± 10.11 (47–91)	− 1.697	0.100
Fathers	Pre-test (first day)	70.53 ± 9.36 (55–91)	− 2.840	0.008*
	Post-test 1 (day 7)	70.40 ± 8.71 (56–90)		
	Post-test 2 (day 15)	69.23 ± 8.99 (51–88)	− 1.567	0.128
Parent total	Pre-test (first day)	71.16 ± 10.04 (51–95)		
	Post-test 1 (day 7)	71.08 ± 9.74 (52–95)	− 3.453	0.001**
	Post-test 2 (day 15)	70.05 ± 9.52 (47–91)	− 2.279	0.026*

^a^ Paired sample t test * *p* 0.05 **p 0.001.

No statistically significant difference was determined between the pre- and post-training mean scores of mothers (*p* > 0.05). While there was a statistically significant difference between the pre- and post-training mean scores of fathers at the first evaluation (post-test 1) (*p* < 0.05), no statistically significant difference was found at the second evaluation (post-test 2) (*p* > 0.05).

A statistically significant difference was found between the mean scores of the parents before and after the training in both the first evaluation (post-test 1) and the second evaluation (post-test 2) (*p* < 0.05).

The mean scores of newborn crying duration before and after IC training are shown in Table 3.

**Table 3:** Comparison of neonatal crying duration before and after IC training (n = 30).

Total crying time (hours)		Mean ± SD (min–max)	t	*p*
First day	Mothers	4.46 ± 1 (3–6)	− 1.099	0.276
	Fathers	4.76 ± 1.1 (3–6)		
Day 7	Mothers	1.76 ± 0.62 (1–3)	− 0.905	0.369
	Fathers	1.91 ± 0.65 (1–3)		
Day 15	Mothers	1.10 ± 0.33 (0.5–2)	− 1.253	0.215
	Fathers	1.21 ± 0.38 (0.5–2)		

t: Student’s t-test *p <  0.05 **p <  0.001.

There was no statistically significant difference between the total mean scores of duration of neonatal crying time recorded by mother and father before and after IC training (*p* > 0.05).

The comparison of the mean crying duration of the newborns before and after the IC training and the effect sizes are shown in Table 4.

**Table 4:** Comparison of mean crying duration of newborns before and after IC training and effect sizes (n = 30).

Total crying time (hours)	Pre-test (first day)	Post-test 1 (day 7)	Post-test 2 (day 15)	Freidman/p	*f*
	Mean ± SD (min–max)				
Mothers	4.46 ± 1 (3–6)	1.76 ± 0.62 (1–3)	1.10 ± 0.33 (0.5–2)	56.857 0.000**	**2.076**
Fathers	4.76 ± 1.1 (3–6)	1.91 ± 0.65 (1–3)	1.21 ± 0.38 (0.5–2)	57.150 0.000**	**1.837**

Freidman **p  0.001 *f*: Cohen’s f.

When repeated measures at different time points were compared, it was found that the mean crying duration of newborns recorded by mothers and fathers decreased over time, and the difference was statistically significant. In addition, these differences were found to have a large effect size. This effect was considered to be clinically significant (Table 4).

## Discussion

IC is a condition that can begin in the neonatal period and last up to 3–4 months, and can cause various problems for both the infant and the parents^6,7^. The most prominent definition of IC is uncontrollable crying. While crying spells cause behavioral problems in newborns and infants^22^, they cause problems such as fatigue, insomnia, stress, and inadequate coping, etc. in parents^11,17^, and these conditions can lead to inadequate breastfeeding and caregiving problems. This study evaluated the effectiveness of training given to parents of newborns diagnosed with IC. It was found that a decrease in the average ICS score occurred after the training and that it had a positive effect on the newborns. It was observed that the joint evaluation of fathers and parents made a difference in this decrease. No study was found in the literature that assessed the effect of parent education on the IC level. El-Bahnasawy et al.^27^ reported a greater improvement in mothers’ IC practices and knowledge after training. The fact that the majority of mothers were housewives in the postpartum period may have caused difficulties in infant care. Fathers who spent less quality time with the baby due to their employment and provided supportive care to the mother may have been effective in IC management in terms of overall father and parent involvement. Due to the paucity of studies on the content of education, the results of studies on practices and risk factors for IC that differ from the method of this study are included. Hannula et al.^22^ reported that reflexology reduced colic problems in a study conducted with parents and their infants. Hikita et al.^11^ examined IC risk factors and treatment methods used by families starting in the neonatal period and reported that parents need more information about IC management. Radwan et al.^2^ investigated the factors affecting infants with IC and reported that the symptoms of IC were more frequent as the infant’s birth month decreased. In this case, it can be interpreted that the management of neonatal infantile colic is more difficult. It can be interpreted that in the neonatal period, the mother needs more support in caring for her baby diagnosed with IC, and her coping skills should be supported more when she is alone with the baby’s problems. It is assumed that this responsibility falls on the father, who is the closest person in the family.

IC causes crying episodes at certain time intervals at a level that negatively affects the lives of both infants and parents^3^. To reduce the frequency and duration of these episodes, infant crying should be controlled^15,31^. According to the results of this study, it was found that the duration of newborn crying decreased after the training. There is no study in the literature that evaluates the effectiveness of training given to parents on the duration of crying caused by IC. It was noted that studies generally evaluated the effects on crying duration after an intervention. The duration of crying in infants with IC was discussed with the results of studies conducted differently from the method of this study. Ateş Beşirik and Geçkil^31^ applied therapeutic touch three days a week, six times a week, in addition to usual care for 2 weeks to 4–12-week-old infants diagnosed with IC and compared IC level, crying, and sleep duration with the control group. The results reported that therapeutic touch effectively reduced symptoms, decreased crying time, and increased sleep duration in infants with IC^31^. Sezici and Yiğit^24^ performed rocking and white noise applications on 40 babies diagnosed with IC in the neonatal period. They reported that playing white noise to colicky babies reduced their daily crying time and increased their sleep time compared to rocking^24^. Castejón-Castejón et al.^23^ performed craniosacral massage on 58 colicky infants aged 0–84 days. They reported that crying time was shorter, sleep duration was longer, and colic severity was lower in the massage group. Landgren and Hallström^21^ reported that acupuncture reduced crying time in infants with IC. Icke and Genc^19^ reported that foot reflexology applied to infants with IC significantly reduced IC scale scores. Ertem and Özyazıcıoğlu^18^ compared massage and rocking methods in infants with infantile colic and reported that the massaged infants cried less, slept more, and had less colic severity. Studies have demonstrated the effects of non-pharmacological methods on the severity of IC, crying duration, and sleep duration. The training provided in this study informed parents that the use of these practices could have a positive effect on IC and reduce crying time in infants. As a result of the study, it was found that the training given to the parents reduced the severity of colic and shortened the crying time. Based on this, it can be interpreted that teaching parents nursing practices and the practices that parents will perform individually or in combination will facilitate the IC method.

### Strengths and limitations

This is one of the few studies on the management of neonatal infantile colic. Family education about infantile colic is a strength of this study, as it provides comfort to both the baby and the family. The fact that there was no control group in the study and that the sample was not randomly selected may affect the results. The fact that mothers did not fully recover after normal or cesarean delivery may have caused them not to focus enough on the training given. These results are specific to the newborns diagnosed with infantile colic and their parents in the sample and cannot be generalized to all newborns with infantile colic and their parents.

## Conclusion

As a result of the evaluation after the IC training, it was found that IC symptoms decreased in newborns. Although it was found that the symptom reduction in mothers did not make a difference in terms of parents, significant differences were found in the first evaluation of fathers after the training and in both the first and second evaluations of parents together.

A significant decrease in newborns’ crying duration due to IC was found after training. However, there was no difference in maternal and paternal ratings. When the mean crying times were compared in terms of maternal and paternal ratings, it was found that these decreases were significantly different and had a high effect value. This finding was considered clinically significant. According to these results, it can be recommended that parents should receive training in neonatal IC together, that the training should start during pregnancy, that parents should be given training booklets, brochures, etc. for correct information, that measurements should be repeated in the following months, and that the study should be repeated with a larger sample in terms of crying time.

## Supplementary Information

Below is the link to the electronic supplementary material.

Supplementary Material 1

Supplementary Material 2

## Data Availability

All data has been provided within the manuscript. The dataset supporting the conclusions of this article is available from the authors upon reasonable request.
