# Supplementary material for: The effect of infantile colic training given to parents on the neonatal infantile colic level and crying duration
Source: Sci Rep. 2026 Jan 28;16:4233. doi: 10.1038/s41598-025-34344-1 (PMC12859028; doi:10.1038/s41598-025-34344-1)
Supplement: Supplementary file 1 — Supplementary Material 1 [file 41598_2025_34344_MOESM1_ESM.docx]

**SOCIODEMOGRAPHIC DATA FORM**

1. Mother Age:…………………
2. Father Age:………………….
3. Mother's Educational Status:

Literate ( ) Primary School ( ) Middle School ( ) High School ( ) University ( )

1. Father's Educational Status:

Literate ( ) Primary School ( ) Middle School ( ) High School ( ) University ( )

1. Is the Mother working? No ( ) Yes ( )
2. Is the Father working? No ( ) Yes ( )
3. What is your family type?

Nuclear Family ( ) Extended Family ( ) Separated Family ()

1. Your baby's Gender: Female ( ) Male ( )
2. Your baby's birth weight:……….gr Height:………cm Head circumference:…… cm
3. Your baby's current weight:……….gr Height:………cm Head circumference:…. cm
4. At which week of your pregnancy was your baby born?:………………
5. The delivery method for this baby: Normal Delivery ( ) Cesarean Section ( )
6. What is the birth order of this baby (How many children do you have in total)?: …
7. How is your baby being fed?

Breast milk only ( ) Breast milk + Water ( ) Breast milk + Formula ( ) Formula only ( )

1. Does the baby use a bottle? Yes ( ) No ( )
2. If the baby uses a bottle, what is the holding position? Horizontal ( ) Vertical ( )
3. Does the baby use a pacifier/dummy? Yes ( ) No ( )
4. How many times a day do you breastfeed your baby?: ………………..
5. What is your baby's daily number of defecations (poops)?: ……………………..
6. Do you apply any method to relieve colic pain? No ( ) Yes ( )

If Yes, what?………………………………..………………………………………

1. On average, how many hours a day does the baby cry?:………………………….
