# Supplementary material for: The effect of infantile colic training given to parents on the neonatal infantile colic level and crying duration
Source: Sci Rep. 2026 Jan 28;16:4233. doi: 10.1038/s41598-025-34344-1 (PMC12859028; doi:10.1038/s41598-025-34344-1)
Supplement: Supplementary file 2 — Supplementary Material 2 [file 41598_2025_34344_MOESM2_ESM.docx]

**INFANT COLIC SCALE**

|  | **I strongly disagree** | **I mostly disagree** | **I somewhat disagree** | **I somewhat agree** | **I mostly agree** | **I strongly agree** |
| --- | --- | --- | --- | --- | --- | --- |
| **Cow’s Milk/Soy Protein Allergy/İntolerance** |  |  |  |  |  |  |
| What Mom eats affects whether or not the baby has colic. |  |  |  |  |  |  |
| What Mom eats affects how bad the colic is. |  |  |  |  |  |  |
| **Immature Gastrointestinal System** |  |  |  |  |  |  |
| Baby does not usually vomit. |  |  |  |  |  |  |
| Baby may still be vomiting when time for the next feeding. |  |  |  |  |  |  |
| Baby vomits milk that looks like it did before it was drunk. |  |  |  |  |  |  |
| Baby has no difficulty passing stool. |  |  |  |  |  |  |
| **Immature Central Nervous System** |  |  |  |  |  |  |
| Baby is jittery. |  |  |  |  |  |  |
| Colic occurs when the baby has had a busy day. |  |  |  |  |  |  |
| Baby does not need to be rocked to sleep. |  |  |  |  |  |  |
| Colic is not related to baby being tired. |  |  |  |  |  |  |
| Baby eats at the same time every day. |  |  |  |  |  |  |
| Baby can go to sleep by himself/herself. |  |  |  |  |  |  |
| Baby is always in motion when awake. |  |  |  |  |  |  |
| Baby sleeps at different times every day. |  |  |  |  |  |  |
| **Difficult İnfant Temperament** |  |  |  |  |  |  |
| Baby is cranky most of the time. |  |  |  |  |  |  |
| Baby does not cry easily. |  |  |  |  |  |  |
| Baby is happy most of the time. |  |  |  |  |  |  |
| Baby waits calmly while I get the food ready. |  |  |  |  |  |  |
| **Parent-İnfant İnteraction Problem İnfant** |  |  |  |  |  |  |
| When baby starts to fuss, nothing I do helps. |  |  |  |  |  |  |
| When the colic starts, I can soothe him/her. |  |  |  |  |  |  |
| When the colic starts, nothing I do helps. |  |  |  |  |  |  |
| I can tell what baby wants when he/she starts to cry |  |  |  |  |  |  |
